# Subcutaneous Ketamine in Depression: A Systematic Review

**DOI:** 10.3389/fpsyt.2021.513068

**Published:** 2021-05-28

**Authors:** Vitor Breseghello Cavenaghi, Leandro Paulino da Costa, Acioly Luiz Tavares Lacerda, Edson Shiguemi Hirata, Eurípedes Constantino Miguel, Renério Fraguas

**Affiliations:** ^1^Department and Institute of Psychiatry, University of São Paulo Medical School, São Paulo, Brazil; ^2^Programa de Transtornos Afetivos, Laboratório Interdisciplinar de Neurociências Clínicas, Department of Psychiatry, Federal University of São Paulo, São Paulo, Brazil; ^3^University Hospital, University of São Paulo, São Paulo, Brazil

**Keywords:** ketamine, depression, antidepressant, glutamate, subcutaneous

## Abstract

**Background:** Ketamine has been shown to produce a rapid and robust antidepressant effect. Though numerous routes of administration have been studied, subcutaneous (SC) has proven to be a convenient and cost-effective route making its use particularly relevant in developing countries. Here we provide a systematic review covering the use of SC racemic ketamine and esketamine in depression, including its efficacy, safety and tolerability.

**Methods:** A systematic literature search was carried out, from inception through March, 2021, using PubMed/MEDLINE, EMBASE and Web of Science, with no limits of language. After identifying 159 potentially relevant articles, 12 articles were selected after applying our inclusion/exclusion criteria. These comprised two randomized clinical trials, five case-reports and five retrospective studies. Given the small number of studies found and their heterogeneous nature, a meta-analysis was not considered appropriate. Here we provide a synthesis of these data including participant characteristics, dose range, efficacy, safety/ tolerability. Risk of bias was accessed using the Cochrane risk of bias tool.

**Results:** SC Ketamine was administered to unipolar and bipolar patients a single or multiple doses, weekly or twice-weekly, a dose-titration approach was made in major studies, dose ranged from 0.1 to 0.5 mg/Kg of racemic ketamine and 0.5–1 mg/Kg of esketamine. Across all studies, SC ketamine showed a rapid and robust antidepressant effect, with response/ remission rates from 50 to 100% following both single or multiple doses, with transitory side effects.

**Conclusion:** SC racemic ketamine and esketamine in depression is a promising strategy showing beneficial efficacy and tolerability. Future studies exploring the SC route, its cost-effectiveness, and a direct comparison with IV and intranasal (IN) protocols are warranted.

**Systematic Review Registration:** CRD42019137434

## Introduction

Ketamine has been studied and used for psychiatric purposes for over 20 years (1). Its rapid and robust antidepressant effect has been reproduced across numerous studies by significantly decreasing the severity of depression, achieving substantial rates of response and remission even for patients that were non-responsive to previous treatments (1–5).

Over the years, interest in ketamine, its racemic compound and enantiomers [i.e., S-ketamine (esketamine) (6) and R-ketamine (arketamine) (7)], has increased in the literature since 2016. This led to FDA approval of intranasal (IN) esketamine for treating depression in 2019 (8, 9).The pharmacokinetic features of ketamine allow for its administration by numerous routes including intravenous (IV) (1–5), subcutaneous (SC) (10), intranasal (IN) (8, 9), oral (11), sublingual (12), and intramuscular (IM) (13). That said, the optimal route of administration has yet to be defined.

Although the efficacy of ketamine for treatment resistant depression (TRD) has been demonstrated using different routes of administration, most studies have adopted the IV, and more recently IN route. The classical IV protocol infuses a dose of 0.5 mg/kg of racemic ketamine over 40 min (1). This protocol requires the use of an infusion pump as well as skilled nursing staff and extended medical supervision, resulting in relatively elevated costs across both infrastructure and human resources. In comparison, IN esketamine does not require an infusion pump and requires less resources, but has an estimated cost of between U$5,664 and 8,142 for the first month of treatment (14).

The SC route has been proposed to be a more convenient, cheaper and less complex route of administration and has been suggested to be as effective as and possibly safer than the IV administration (6). This route has also been used to treat other conditions since 1975 (15).

Although there is still controversy regarding an exact dose-response relationship, both dosage and route of administration directly influence the efficacy and tolerability of ketamine and its enantiomers. SC is an easier and more convenient route, not requiring equipment and skilled staff. This is especially relevant in developing countries that struggle to optimize scarce resources. The main aim of this systematic review is to assess the efficacy, tolerability and feasibility of SC ketamine and its enantiomers for the treatment of depression.

## Methods

The study protocol was registered on the International Prospective Register of Systematic Reviews database (PROSPERO; registration number: CRD42019137434) and adheres to the Preferred Reporting Items for Systematic Reviews and Meta-Analyses (PRISMA) guidelines (16).

### Search Strategy and Data Sources

A systematic literature search was carried out using PubMed/MEDLINE, EMBASE and Web of Science, from inception up until March 12, 2021. The search terms employed were ketamine AND subcutaneous AND depress^*^.

### Studies Selection and Data Extraction

After excluding duplicates, two authors (VC and LC) independently reviewed the abstracts to check eligibility. Initial selected articles were retrieved in full text to apply inclusion/ exclusion criteria and confirm eligibility. Disagreements were discussed with a third author (RF) and resolved by consensus.

### Inclusion/Exclusion Criteria

To be considered eligible, each accepted study must had included patients with a diagnosis of Major Depressive Disorder or Major Depressive Episode (unipolar or bipolar). SC racemic ketamine, esketamine or arketamine must have been used in at least one session. Studies using ketamine associated with other interventions such as ECT were considered ineligible. We accepted clinical trials, retrospective studies, and case reports. We accepted all languages and there were no limits regarding age of participants.

### Data Analysis and Summary Measures

The above criteria identified 12 acceptable studies. Given that we found so few studies with heterogeneous methods, a meta-analysis was not considered appropriate and a narrative review was performed.

Thus, here we provide a synthesis of these data including characteristics of participants, dosing, study design, and findings. Table 1 (Risk of bias) was made employing Cochrane risk of bias tool (19), Table 2 (Mood outcome) included a summary of the following characteristics: methods, participants, number of subjects, dose, number of sessions, remission and response, while Table 3 (Safety and Tolerability assessment) the tools employed by each study to assess Safety and Tolerability were spared in the following categories: psychiatric or psychotomimetic, neurological or cognitive, cardiovascular, other.

**Table 1:** Risk of bias.

	**Random sequence generation**	**Allocation concealment**	**Blinding of participants and personnel**	**Blinding of outcome assessment**	**Incomplete outcome data**	**Selective reporting**	**Other bias**
George et al. (17)	Low risk	Low risk	Low risk	Unclear	Low risk	Low risk	Low risk
Loo et al. (18)	High risk	High risk	To placebo low risk, to routes high risk	Unclear	Low risk	Low risk	Low risk

*Risk of bias assessment based on modified Cochrane risk of bias tool*.

**Table 2:** Mood outcome.

**References**	**Method**	**Participants**	**N**	**Dose**	**Number of sessions**	**Remission**	**Response**
George et al. (17)	Phase 1 (blinded) was followed by a phase 2 (open label) with 8 sessions.	Patients ≥ 60 years and MDD or BP. ≥1 treatment without response in the current episode.	16	Ketamine at 0.1–0.5 mg/kg. Titrated by 0.1 mg/kg if no response.	First phase: 1 to 5, dosed weekly. Second phase 12 sessions, first 8, dosed twice-weekly, next 4, weekly	Phase 1 (RCT): 7 in 16 patients = 43.75% Phase 2 (open label): 2 in 7 patients = 28.5% -9 from 16 patients remitted at least at 1 end-point = 56.25%	Phase 2 (open label) 4 from 7 = 57% of responders -11 from 16 patients met response at least at 1 end-point = 68.8%
Loo et al. (18)	Patients were assigned to IV, IM or SC injection. Active placebo (midazolam) randomly inserted among 3 first applications.	Patients ≥ 18 years and MDD/. ≥1 adequate trials of an antidepressant.	15	Ketamine at 0.1–0.5 mg/kg. Titrated by 0.1 mg/kg if no response.	1 to 5, dosed at least 1 week apart	Response/remission rates of 75% (IV), 60% (IM) and 100% (SC).	Response/remission rates of 75% (IV), 60% (IM) and 100% (SC).
Iglewicz et al. (20)	Retrospective study, 2 patients received a single dose of SC ketamine.	31 inpatients at a hospice care with depression, aging from 44 to 89 years.	2	Ketamine at 0.5 mg/kg	1 received a single oral dose followed by a SC, and 1 received a single SC dose.		General improvement in CGI. No distinction was made between oral and SC ketamine.
Gálvez et al. (21)	Patient from the clinical trial above.	55 years old female with melancholic depression.	1	Ketamine at 0.1–0.2 mg/kg	First phase: 2, dosed weekly. Second phase: 12, first 8, dosed twice-weekly, next 4, weekly	First phase: remission after 0.2 mg/kg single dose Second phase: remission after 12th dose	First phase: response after 0.1 mg/kg single dose Second phase: response after 3rd dose
McNulty and Hahn (22)	Single SC treatment	A palliative care patient 44 year old patient with depression, anxiety and chronic pain.	1	Ketamine at 0.5 mg/kg	1		Dramatic relief from pain, anxiety, and depression for 80 h
Costa et al. (23)	Single SC treatment.	75 years old patient, bipolar depression.	1	Esketamine at 0.5 mg/kg	1	MADRS ranged from 20 to 2 after 24 h	
Barbosa et al. (24)	Progressive dosage of esketamine.	65 years old patient, metastatic cancer in palliative care.	1	Esketamine 0.5 mg/kg (first); 0.75 mg/kg (2nd−4th)	4, dosed twice-weekly	Clinical remission after 3 sessions.	
Rocha et al. (25)	Progressive dosage of esketamine, 0.5, 0.75, and 1 mg/kg, increased if the patient did not respond to the previous dosage.	76 years old patient with Alzheimer Disease and Epilepsy.	1	Esketamine 0.5 mg/kg (first); 0.75 mg/kg (2nd−3rd)	8, dosed twice-weekly		Improvement in general state, CGI-I ranged from 8 to 1, clinically remitted
Lucchese et al. (26)^*^	Progressive dosage of esketamine, 0.5, 0.75, and 1 mg/kg, increased if the patient did not respond to the previous dosage.	Patients with MDD or BD, ≥ 15 years old; ≥2 adequate trials of an antidepressant, MADRS ≥ 25.	70	Esketamine at 0.5, 0.75, or 1 mg/kg.	6, dosed weekly		50% of response

* *The sample is the same of Fava et al. (27), Delfino et al. (28), and Del Sant et al. (29)*.

**Table 3:** Safety and tolerability assessment.

	**Psychiatric or psychotomimetic**	**Neurological or cognitive**	**Cardiovascular**	**Other**
George et al. (17)	BPRS, YMRS, CADSS	SAFTEE	Heart rate, blood pressure	SAFTEE
Loo et al. (18)	BPRS, YMRS, CADSS	SAFTEE	Heart rate, blood pressure	SAFTEE, liver function
Iglewicz et al. (20)	CGI	CGI	/	CGI
Gálvez et al. (21)	BPRS, YMRS, CADSS	SAFTEE	Heart rate, blood pressure	SAFTEE
McNulty and Hahn (22)	/	/	/	/
Costa et al. (23)	BPRS, YMRS, CADSS	MOAA/S^1^	Heart rate, blood pressure. oximetry	/
Barbosa et al. (24)	/	/	Heart rate, blood pressure, oximetry	Visual Analogical Scale (VAS) for Pain
Rocha et al. (25)	CGI	CGI	Heart rate, blood pressure, oximetry	CGI
Del Sant et al. (29)^*^	/	/	Heart rate, blood pressure, oximetry, respiratory rate	/

* *The sample is the same of Fava et al. (27), Delfino et al. (28), and Lucchese et al. (26)*.

1 *Modified observer's assessment of alertness/sedation scale*.

### Risk of Bias in Individual Studies

The risk of bias was accessed by two authors using a risk of bias assessment based on a modified Cochrane risk of bias tool (19). Disagreements were discussed with a third author and resolved by consensus. Each study received a score of low, high or unclear risk of bias in each category.

## Results

### Studies Selection

**Figure 1:**
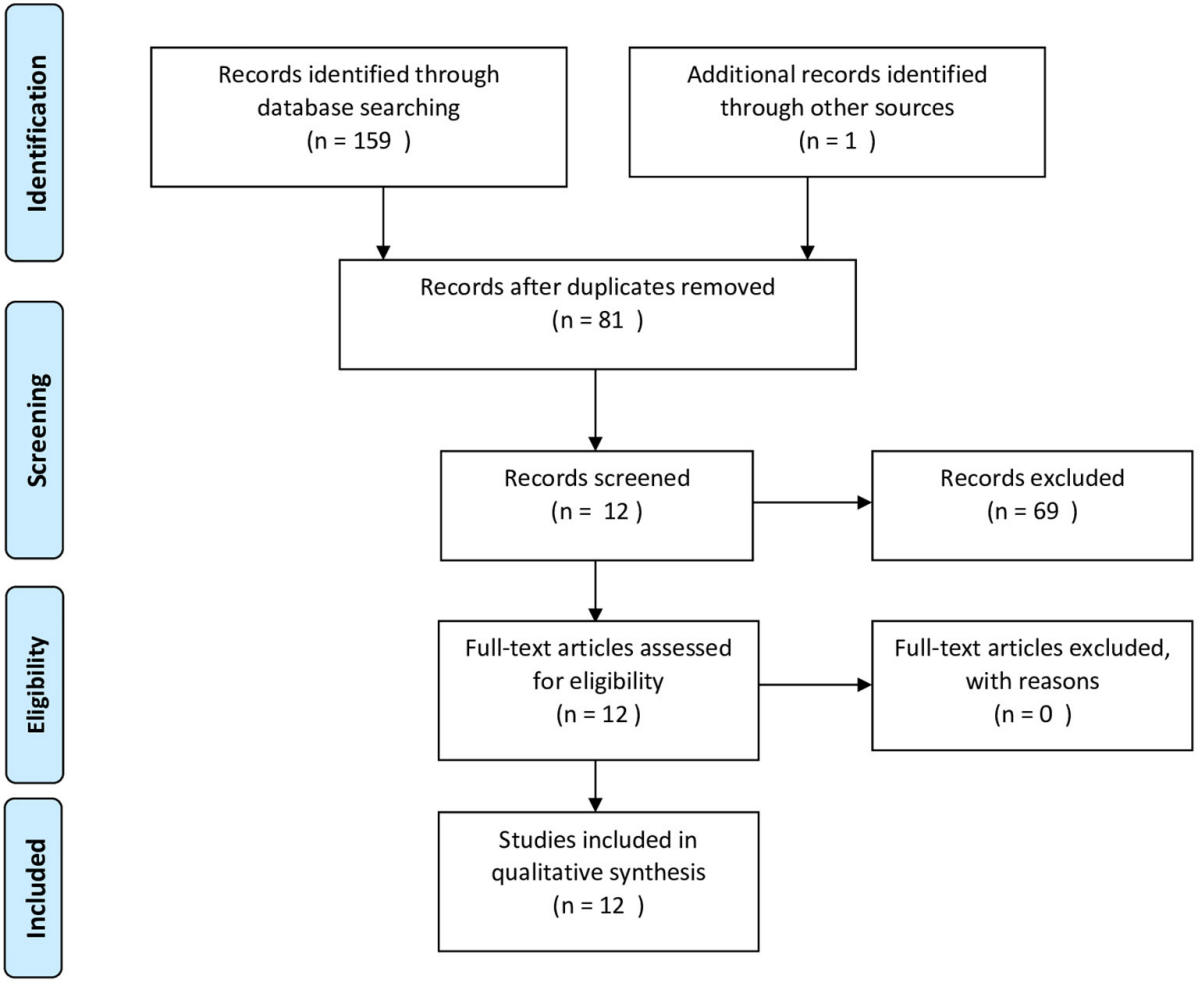
PRISMA flow diagram of search strategy.

One hundred fifty-nine studies were found via our electronic database search: Pubmed/MEDLINE (*n* = 59), EMBASE (*n* = 53) and Web Of Science (*n* = 47). Eighty articles were selected after removing duplicates and abstracts were accessed. Eleven articles were selected after removing studies that did not meet inclusion/ exclusion criteria and were retrieved in full text. One article was retrieved from the references section of one identified study. Thus, 12 articles are included in this review (Figure 1).

### Risk of Bias Within Studies

Details of the risk of bias assessment are provided in Table 1, it was excluded from this assessment case reports and chart review. The study conducted by Loo et al. (18) was classified as low risk of bias for allocation concerning active placebo vs. ketamine. However, no mention was made regarding strategies for blinding the route of administration, IV, IM or SC, which increases the risk of bias.

### Synthesized Findings

Of the selected articles, two were randomized clinical trials (17, 18) five were case-reports (21, 22) and five were retrospective studies (20, 26–29) (see Table 2). From the retrospective studies, four of them (26–29) were from the intervention in the same group of patients and, thus, will be presented together in Tables 2, 3.

### Mood Outcome

In a randomized double-blind multiple-crossover placebo-controlled trial followed by an open label phase, George et al. (17) enrolled 16 unipolar or bipolar depressed patients aged > 60 years. Patients showed an insufficient response to at least one treatment during the current mood episode and had MADRS ≥ 20. All patients remained on a stable dose of psychotropic medications during the study. In the randomized clinical trial (RCT) phase, patients received weekly SC racemic ketamine at a progressive dose, starting at 0.1 mg/kg and increasing 0.1 mg/kg, each session, up to 0.5 mg/kg. The protocol was stopped when the patient reached remission (MADRS <10) at day 7 after last administration or at the fifth session, with 0.5 mg/kg. For non-remitters, the dose was increased up to 0.5 mg/kg. Seven participants (43,75%) met the criterion for remission, 1 week after last administration (ketamine doses 0.1 mg/kg: *N* = 1; 0.3 mg/kg: *N* = 1; 0.4 mg/kg: *N* = 3; and 0.5 mg/kg: *N* = 2) and were followed until relapse. Seven participants (43.75%) received up to 0.5 mg/kg and did not remit. Two patients dropped out due to unrelated illness. MADRS scores significantly decreased for 0.2 mg/kg (*p* < 0.01), 0.3 mg/kg (*p* < 0.001), and 0.4 mg/kg (*p* < 0.001) doses, but not for the 0.1 mg/kg (*p* = 0.06) dose, as compared with placebo (midazolam). In the open label phase, patients received 12 administrations of the dose of remission or 0.5 mg/kg if remission was not attained with lower doses. In this phase, 12 patients, 5 RCT remitters who relapsed and 7 RCT non-remitters, received SC racemic ketamine twice weekly for 4 weeks and then weekly for 4 weeks. For the seven RCT non-remitters, two attained remission with repeated treatments (0.5 mg/kg).

Loo et al. (18) conducted a double-blind, placebo-controlled trial comparing routes of administration of racemic ketamine: IM (*N* = 5), IV (*N* = 4) and SC (*N* =6). They included 15 patients aged > 60 years with a diagnosis of major depression disorder (MDD), a MADRS ≥ 20 and an insufficient therapeutic response to at least one antidepressant trial. The patients were allowed to maintain psychotropic medications in stable doses during the protocol. The protocol used a progressive ketamine dose ranging from 0.1 to 0.5 mg/kg, with intervals of at least 1 week and increments of 0.1 mg/kg a week, if the patient did not reach remission by the 7th day (MADRS <10). The use of IV ketamine was double-blinded with administration of active placebo (midazolam) at 0.01 mg/kg. Twelve patients reached remission [75% (IV), 60% (IM) and 100% (SC)] and all three routes of administration resulted in comparable antidepressant effects.

We identified two case reports examining response to SC racemic ketamine (21, 22). In one case (22), a single dose of 0.5 mg/kg significantly reduced symptoms of depression and anxiety. In the other case (21), the patient showed a remission for 5 months after 2 doses (0.1 and 0.2 mg/kg), dosed at least a week apart. She then received 12 more doses (first 8, dosed twice-weekly, next 4, weekly) of 0.2 mg/kg and remained remitted for 10 weeks after the last injection.

We identified three case reports using SC esketamine. In the first report of SC esketamine, Costa et al. (23) reported remission of symptoms in a 75 year-old patient with bipolar depression [who was resistant to electroconvulsive therapy (ECT)] after a single session of SC esketamine at 0.5 mg/kg. Barbosa et al. (24), in a setting of palliative care, reported a case of remission after 3 sessions (of a total of 4, dosed twice-weekly) with esketamine from 0.5 to 0.75 mg/kg. The third case report administered SC esketamine in a 76 year-old patient with depression and Alzheimer Disease. The patient showed improvement in depressive symptoms - dose ranged from 0.5 to 0.75 mg/kg in three sessions (eight in total, dosed twice-weekly) (25).

The identified chart review (20) reported two patients (among a group of 31 patients) that received a single dose of SC racemic ketamine at 0.5 mg/kg. Unfortunately, the results were not presented separately from other patients (*n* = 29) who had received oral ketamine. The entire group (*n* = 31) showed significant global improvement as measured using the Clinical Global Impression (CGI).

In a retrospective real world study, Lucchese et al. (26) described 70 patients aged > 15 years with a diagnosis of MDD (39 patients) or BD (31 patients), who showed no response to ≥ 2 treatment in a current episode and a MADRS ≥ 25. Patients received six sessions of SC esketamine, dosed weekly, starting with dosage of 0.5 mg/kg. The dosage was increased by 0.75 or 1 mg/kg if the patient did not respond to the previous dosage (decrease in MADRS by ≥ 50%) 1 week later. Eighty percentage of patients had not responded to five previous antidepressant treatments and the current episode was longer than 2 years for 70%. Fifty one (72.9%) patients had dosage titrated to 1 mg/kg of esketamine. Thirty-five (50%) patients responded. Anhedonia was evaluated separately by item 8 of the MADRS (inability to feel), and improvement was observed 24 h after the first session (*t* = 4.007; *p* < 0.001), with a further reduction in anhedonia scores following repeated infusions. No significant differences were observed between MDD and BD (28).

In this same group of 70 patients, the probability of response after each of the first four sessions of esketamine was estimated by hidden Markov modeling. The probability of a non-responder to become a responder after an injection was 17.30%, while the probability for a “responder” to remain as a “responder” was 95% (27).

### Safety and Tolerability of SC Route

Loo et al. (18) and George et al. (17) assessed tolerability with positive symptom items from the Brief Psychiatric Rating Scale (BPRS) (30), item 1 (Elevated mood) of the Young Mania Rating Scale (YMRS) (31) and the Clinician Administered Dissociative Symptoms Scale (CADSS) (32). They took these measurements at baseline, 40 min after injection and after 4 h. Other side-effects were accessed with a modified version of the SAFTEE scale (33). Orientation and simple and complex reaction times were measured at baseline and 4 h after injection. Hemodynamic effects were evaluated with measurement of heart rate and blood pressure at 5, 10, 30, 60, and 240 min after administration. Liver function was also measured by George et al. (17) before and after RCT.

Loo et al. (18) found dissociative psychotomimetic effects directly related to dose. The IV group had higher peak scores when evaluated 40 min after dosage. Symptoms included mild depersonalization, derealization, altered body perception and altered time perception. Items from the BPRS and Item 1 of YMRS did not show mania symptoms at observed endpoints. Other reported side-effects were fatigue, lightheadedness, dizziness, blurred vision and emotional lability. Increases in heart rate and blood pressure did not exceed 120%. The peak of reported effects were between 10 and 15 min, resolving spontaneously between 30 and 60 min.

George et al. (17) also observed a dose–response relationship for dissociative psychotomimetic effects. Symptoms reported were: mild perceptual disturbance (colors or sounds seemed different), derealization, altered body perception, and altered time perception. Peak effects occurred 10–15 min after injection. BRPS and YMRS showed no emergent psychiatric symptoms at any time point and there were no clinically significant changes compared to the midazolam condition. Transient increases in systolic and diastolic blood pressure were occasionally observed, with peak incidence 4 h after administration and increases in heart rate only exceeded 120% four times, but did not exceed 131.5%.

Other side-effects reported were: Palpitations, Flushing, Lightheadedness/ Dizziness, Fatigue/ Sleepiness/ Poor Concentration/ Feeling Vague (Spaced Out), Paresthesia, Nausea, Dry Mouth, Blurred Vision/Diplopia, restlessness, and headache. All reported side-effects resolved within 30–60 min without the need for medical intervention. All participants were oriented at 4 h post-treatment. Liver function from 14 patients was within normal limits except for discrete elevations of transaminases in two patients after RCT.

In the open label phase minimal increases in heart rate, blood pressure, and CADSS scores were observed with no evidence of cumulative increases. Dizziness (*n* = 2), numbness (*N* = 2), headache (*n* = 1), and urge to urinate slightly more often (*n* = 1) were reported and resolved spontaneously. One patient (of eight) showed a slight increment in transaminases after the course.

Iglewicz et al. (20) assessed side effects with ratings on the Clinical Global Impression (CGI) scale based on a palliative care team charting at baseline and post-ketamine dosing. The results from SC ketamine were presented together with an oral dosage and showed that 3 (13.6%) patients had only 1 side effect, 6 (27.3%) had up to three psychiatric side effects and 13 (59.1%) had no side effects. The side effects reported were: disorientation [*N* = 7 (45.5%)], hallucination [*n* = 4 (18.2%)], sedation [*N* = 4 (18.2%)], insomnia [*N* = 1 (4.5%)], delusions [*N* = 1 (4.5%)], and anxiety [*N* = 1 (4.5%)].

Del Sant et al. (29) described in a sample of 70 patients [same from Lucchese et al. (26), Fava et al. (27) and Delfino et al. (28)] that SC esketamine was well-tolerated for doses of 0.5, 0.75, and 1 mg/kg. Systolic Blood Pressure (SBP) increased about 4.87 while Diastolic Blood Pressure (DBP) increased 5.54 mmHg within 30–45 min, returning to baseline within 120 min. No significant heart rate changes were observed. 14/70 patients had SBP > 180 mmHg and/or a DBP >110 mmHg. Other assessments of tolerability in this sample were made but the manuscript is still in preparation by authors.

In the case reports ketamine and esketamine were generally well-tolerated, with reports of mild lightheadedness and blurred vision (21), transient elevation in blood pressure and heart rate (23, 24) and abdominal pain (24).

The tolerability assessment is presented in Table 3. The measurements that were most often employed were the BPRS, YMRS, CADSS, SAFTEE, heart rate and blood pressure.

### Other Findings

The ketamine blood concentration was assessed by Loo et al. (18). Blood samples were obtained at baseline, and then 5, 15, 30, 120, and 240 min after IV dosing and 15, 30, 120, and 240 min after IM/SC injection. Plasma concentrations recorded after IV showed a peak between 350 and 400 ng/ml (dose of 0.5 mg/Kg), with a peak below 200 ng/ml in SC route. Plasma concentrations were linearly correlated with the ketamine dosage (IV, *r* = 0.88, *P* < 0.001; IM, *r* = 0.92, *P* < 0.001; SC, *r* = 0.86, *P* < 0.001) as well as CADSS scores at 40 min (*r* = 0.44, *P* = 0.001).

No data were presented regarding costs or cost-effectiveness of the SC route in depression. The only estimated cost was presented by Lucchese et al. (26) of one esketamine ampoule (50 mg/mL, 2 mL) at BRL R$15.00 (~US$2.70) for approximately two dosages.

## Discussion

In this review we found twelve articles examining SC racemic ketamine and esketamine in depression. The results up to now are promising, with efficacy comparable to IV ketamine and only transitory side effects. However, many limitations, such as a relatively small number of patients and patients from the same sample, limit the findings.

The use of SC ketamine has been described in humans since 1975 (15) and has been explored mainly for pain (15, 34–36) perioperative analgesia (37–39) and anesthesia (10). Javid et al. (10) found that the SC and IV routes similarly produced a dissociative consciousness in a laparoscopic procedure. They proposed that the SC route is safer, since some patients in the IV group lost their ability to cooperate and experienced mild hallucinations. The SC dose used, however, was 0.6 mg/kg while the IV protocol appeared to be in bolus. We note that these dosages are not common in most protocols used for depression. In another study (38) comparing IV to SC routes for pain relief after tonsillectomy, at a SC dosage of 0.5 mg/kg for both routes and the IV administration performed in bolus, the results were similar for both routes. For depression, the first trial conducted by Berman et al. (1) used an IV protocol that has been widely replicated in studies addressing the antidepressant effect of ketamine. Their protocol consisted of 0.5 mg/kg of ketamine administered intravenously with an infusion pump over 40 min.

As discussed earlier, the use of an SC route is mainly motivated by ease of use and possible reduction of costs in both equipment and human resources. The cost-effectiveness of ketamine was evaluated in previous studies for traumatic injuries (36) and Chiari disease (39). Despite potentially important cost-benefit advantages associated with the use of an SC route for treating depression, the present review showed that, up to now, only the costs of an esketamine ampoule (50 mg/mL, 2 mL) has been presented in the literature, costing BRL R$15.00 (~US$2.70) for approximately two dosages (26). For comparison, the costs estimated for IN esketamine range from U$5,664 to 8,142 for the first month of treatment, while IV racemic ketamine costs in the United States range from U$500 to 1,000 per session (14). This cost would be prohibitive in developing countries.

In this review, we found encouraging results for the SC use of ketamine in the treatment of depression. Favorable results regarding both efficacy and safety were reported in case reports (21–25). Data from retrospective studies (20, 26–29) and two clinical trials (17, 18) also confirmed efficacy and a solid tolerability profile associated with SC administration of ketamine for depression. Considering the paucity of data, we will discuss efficacy and tolerability of these results qualitatively, keeping in mind the limited nature of the data at hand.

### Efficacy

To date, the available data support the efficacy of the SC route. Loo et al. (18) demonstrated that all patients who received SC ketamine showed remission or response (100%) at least at one endpoint with a dosage below 0.5 mg/kg. Despite the small number of patients, the results were impressive. In the dose titration study conducted by George et al. (17) involving elderly patients with MDD and BD, 11 (68.8%) of 16 patients responded and nine (56.25%) remitted, some with a dosage even lower than 0.5 mg/kg. For comparison, we note that two previous studies using the IV route reported remission rates of 23% (5) and 29% (2). These data are comparable with results of other studies using the IV route: 50% of response (1), 71% of response and 29% of remission (2), 64% of response (3), 59% of response and 23% of remission (5). One study using IN esketamine documented a 69.3% rate of response (9) Thus, despite the paucity of available data, results found with SC ketamine and esketamine are promising.

Our ability to evaluate the dose-response effect of the SC route measured in the two trials with ketamine, as well as the four retrospective studies (26–29) and two case-reports (24, 25) with esketamine in comparison to IV studies is limited since the latter studies used fixed doses. However, findings from dose-titration studies appear promising with patients remitting following low doses. This will be important for patients that are vulnerable to side effects, including the elderly and those with medical comorbidities. Dose titration was also used in an IV racemic ketamine protocol (4), with doses of 0.1, 0.2, 0.5, and 1 mg/kg. Results indicated no clear or consistent efficacy for the dosages of 0.1 and 0.2 mg/kg.

### Safety and Tolerability

Loo et al. (18) and George et al. (17) used similar parameters to assess tolerability [except for the liver function assessed in the latter (14)], and were in agreement with the literature (40). Loo et al. (18) found better tolerability in the SC group, which had lower scores in dissociative psychotomimetic symptoms (CADSS) as compared to the IV group. However, no significant main effect for any route was found. The IV administration was performed in 2–5 min, which may increase psychotomimetic symptoms. We note that blood concentration was almost double for IV route compared to both SC and IM routes. Blood concentrations using a 0.5 mg/kg IV protocol in 40 min have been reported to be around 200 ng/dL (41) similar to that found for the SC route in the study of Loo et al. (18). Also, the instruments used to assess tolerability were in agreement with the literature (40). Thus, to date, SC ketamine and esketamine demonstrate a solid tolerability profile, with few and transient side effects, similar to the IV (40) and IN routes (8, 9). In addition, considering that two case reports (16, 24) and a retrospective study (20) were performed in the context of palliative care, and another trial involved elderly patients (17), most of the data considered here were from patients with medical comorbidities.

## Future Perspectives

Since the work of Berman et al. (1), many studies have aimed to assess the effect of ketamine in MDD and BD. Since 2016, esketamine has been investigated since it is a more potent antagonist of the NMDA receptor. A recent systematic review showed that racemic ketamine, compared to esketamine, demonstrated greater overall response rates (RR = 3.01 vs. RR = 1.38), remission rates (RR = 3.70 vs. RR = 1.47), as well as lower dropouts (RR = 0.76 vs. RR = 1.37) (42). Knowing the heterogeneity of studies, including that all esketamine studies were IN and ketamine studies were IV, should we return to racemic ketamine or explore esketamine more? Recently, a study using arketamine showed promising effects without dissociative effects (7). The question of whether racemic ketamine or its enantiomers are better for each case remains an open question.

Furthermore, the best route of administration is still an open question more than 20 years later. IV ketamine was the first protocol studied, with all its advantages and disadvantages. IN esketamine appeared to solve some of these problems with practical dispositive and feasibility, leading to its approval by several regulatory agencies throughout the world. However, there is a possible impact on efficacy when using IN esketamine (42). The IM and SC routes appear to be somewhat practical, but as we found in this review regarding SC, the studies in the literature are still few. This review has shown that there remains a lack of a robust trials comparing these routes, or even comparing racemic ketamine to its enantiomers.

## Limitations

Five of the twelve studies we reviewed were case reports, and another five were retrospective studies. In the study conducted by Iglewicz et al. (20), the results for SC ketamine were not described separately and the depression criteria was not clear. Also, in the McNulty and Hahn (22) report, the depression diagnosis criteria was also not clear.

The report by Galvez et al. (21) is a case study of a patient that took part in the study published by George et al. (17). The trials conducted by Loo et al. (18) and George et al. (17) enrolled a small number of patients (15 and 16 patients, respectively). In addition, George et al. (17) studied subjects that were ≥60 years of age, which is not representative of the general population, and patients with BP beyond MDD. In addition, three (17, 18, 21) of five studies were performed on the same group of individuals.

The retrospective studies by Fava et al. (27), Del Sant et al. (29), Delfino et al. (28), and Lucchese et al. (26) were from the same sample and from the same site, which also limits the findings.

## Conclusion

There are scarce data on SC racemic ketamine and esketamine for depression, and there is no study comparing this route with the most commonly used IV or IN protocols. Also, there are no data addressing the cost or cost-effectiveness of this route. The SC route may be particularly appealing to developing countries, where resource scarcity is often a major limiting factor. Available data suggest that SC ketamine and esketamine is a promising alternative for TRD, showing solid efficacy and tolerability. Future randomized clinical trials comparing routes of administration focusing on efficacy, tolerability, pharmacokinetics, cost-effectiveness and long term follow up assessments are needed.

## Data Availability Statement

All datasets generated for this study are included in the article/supplementary material.

## Author Contributions

VC, LC, and RF designed the study. VC and LC conducted literature search and data extraction. Disagreements were discussed with RF and resolved by consensus. VC and LC wrote the first draft. AL, EH, EM, and RF contributed to data interpretation and revised the manuscript critically, contributing to many aspects of the discussion. All authors contributed to and approved the final version of the manuscript and agreed to be accountable for all aspects of the work in ensuring that questions related to the accuracy or integrity of any part of the work are appropriately investigated and resolved.

## Conflict of Interest

AL has received consulting fees from Hoffmann–La Roche, Genentech, Janssen Pharmaceutical, Daiichi Sankyo, Cristalia Produtos Químicos e Farmacêuticos, Pfizer, Mantecorp Indústria Química e Farmacêutica, Libbs Farmacêutica, FQM Farma, and Sanofi-Aventis over the last 24 months and has received research fees from Janssen Pharmaceutical, Eli Lilly, Novartis, Biophytis, Celltrion, Azidus, H. Lundbeck A/S, Servier Laboratories, Hoffman-La Roche, FQM Farma, and Forum Pharmaceuticals. The remaining authors declare that the research was conducted in the absence of any commercial or financial relationships that could be construed as a potential conflict of interest.
